# A Simplified Analysis Method for the Piezo Jet Dispenser with a Diamond Amplifier

**DOI:** 10.3390/s18072115

**Published:** 2018-07-02

**Authors:** Guiling Deng, Na Wang, Can Zhou, Junhui Li

**Affiliations:** 1 The State Key Laboratory of High Performance Complex Manufacturing and School of Mechanical and Electrical Engineering, Central South University, Changsha 410083, China; gldeng@csu.edu.cn (G.D.); nawang@csu.edu.cn (N.W.); LIjunhui@csu.edu.cn (J.L.); 2 School of Information Science and Engineering, Central South University, Changsha 410083, China

**Keywords:** piezo jet dispenser, simulation, diamond amplifier, simplified method

## Abstract

Diamond amplifiers have been widely applied in Nano actuators and Robots. In order to study the dynamic characteristics of the diamond amplifier system which is used in the piezo jet dispenser, it is simplified as a spring-mass-damper system. The dynamic characteristics of the jet dispenser system are analyzed with the simplified method. The characteristics are also tested. The results agree with the simulation, which proves the method is feasible. It will provide a simplified and intuitive representation of the movement of the amplifier, and also provide reliable simulation and experimental platforms for jet dispensing analysis.

## 1. Introduction

Dispensing is a significant technology in the microelectronics packaging industry [1,2,3,4,5,6,7,8]. Piezo jet dispensers have the advantages of small size, high resolution, high frequency, and low-energy consumption, so they have been widely applied [9,10,11,12].

The needle requires 200–300 μm or even greater displacement to achieve better jet performance [13]. The elongation rate of the piezoelectric material is about 0.1–0.15% [14]. The output displacement of the piezo stacks is not large enough for the jet dispenser. A mechanical amplifier is usually applied to improve dynamic characteristics of piezo jet dispenser [15,16,17]. Most designs use flexible hinges. When the jet dispenser works, high stresses in the hinges usually cause damage. To tackle the motion interference issue, a diamond amplifier is proposed [18,19,20].

The mechanical amplifier is an important part of the piezo jet dispenser. Performance of the piezo jet dispenser is determined by the amplifier. It is essential to study the dynamic characteristics of the diamond amplifier for jet dispenser design. Previous research mainly focused on the maximum displacement [21,22,23]. The lumped parameter method was used to analyze the dynamic characteristics of the piezo jet dispenser [16,20], but this method involves a heavy calculation burden.

A spring-mass-damper system simplified method is proposed in this paper, where the diamond amplifier system is simplified as a single or double degree spring-mass-damper system with only a little calculation. The mathematical models and simulation models are established, and the dynamic characteristics are also tested. The tested results agree with the simulation, which proves that the simplified method is reliable. This study will provide a simple approach to designing or assessing a diamond amplifier-based system.

## 2. Experimental System

**Figure 1:**
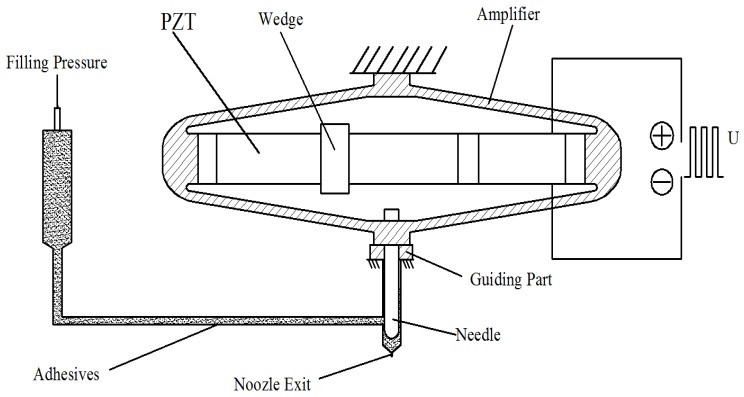
Piezo jet dispenser.

The operating principle of the piezo jet dispenser with a diamond amplifier is the following: adjust the needle to make it come into contact with the nozzle and form a closed chamber. Then, apply pneumatic pressure to the fluid and make it flow into the chamber. A pulse voltage is applied to the piezo stack to cause a vertical stretching motion of the amplifier. Consequently, the needle and the amplifier obtain an up-and down motion. The fluid, driven by the pneumatic pressure, flows into the nozzle. When the needle hits the nozzle, the fluid is rapidly ejected through the nozzle and breaks from it with fluid momentum. The system mainly includes Excitation voltage, PZT, Wedge, Amplifier, Guiding Part, Needle, Filling Pressure, Adhesives, and Nozzle Exit, as shown in Figure 1.

**Figure 2:**
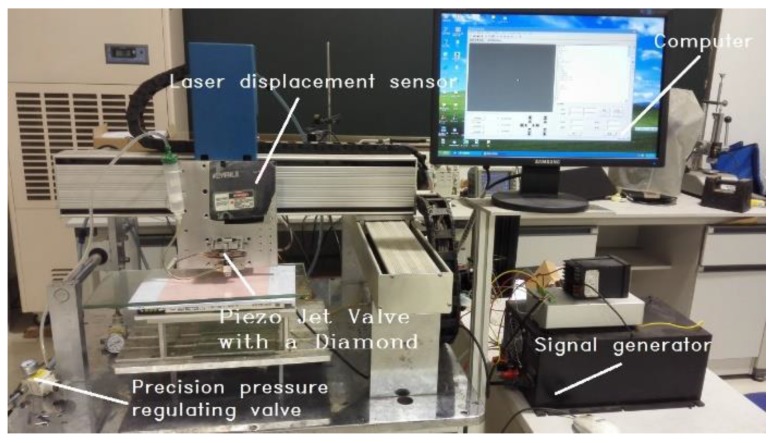
Experimental facilities used to measure the amplifier.

**Figure 3:**
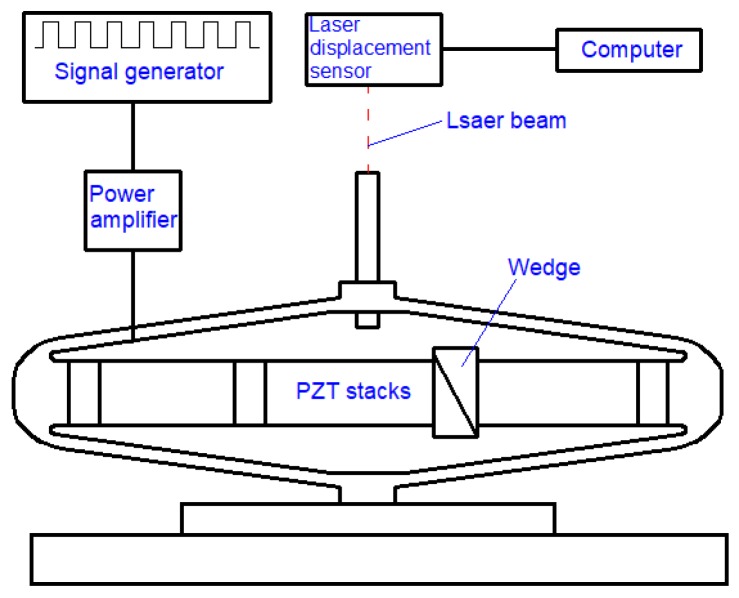
Structure diagram for the amplifier’s experimental facilities.

The experimental system consists of four parts: a piezo jet dispenser, a signal generator, a glue supply system, and a laser displacement sensor system. The experiment facilities used to measure the dynamic characteristics of the amplifier are shown in Figure 2; the structure diagram is shown in Figure 3.

The needle’s displacement is measured by the KEYENCE LK-G laser sensor, whose sampling frequency is 20 KHz. The control signal, generated by an AFG-3102 signal generator from Tektronix, Inc (Beaverton, ON, USA), is amplified by XE-500 power amplifier. As the pulse voltage is supplied to the piezo stacks, the diamond amplifier with the needle is fixed on the table, and a laser sensor is fixed on the top of the needle. When the experimental system works, a laser beam shoots at the needle vertically, and the real-time displacement is tested and recorded.

## 3. Dynamic Models of the Jet Dispenser

The jet dispenser consists of the piezo stacks, a diamond amplifier, and a special injector. To simplify the motion process of the system, a dynamic analysis of the system is carried out.

### 3.1. Mathematical Model of the PZT

Output characteristics of the piezo stacks are mainly determined by input parameters and preload. Performance of piezo stacks are analyzed with different technical parameters. Combined with the knowledge of spring-mass-damper system dynamics analysis, the dynamics analysis of piezo stacks can be carried out.

The material of piezo stacks used in this experiment is PZT5. Material properties are density 7500 kg/m^3^, elastic modulus 36.7 Gpa, Poisson’s ratio 0.32. The technical parameters of each of the piezo stacks are listed in Table 1.

**Figure 4:**
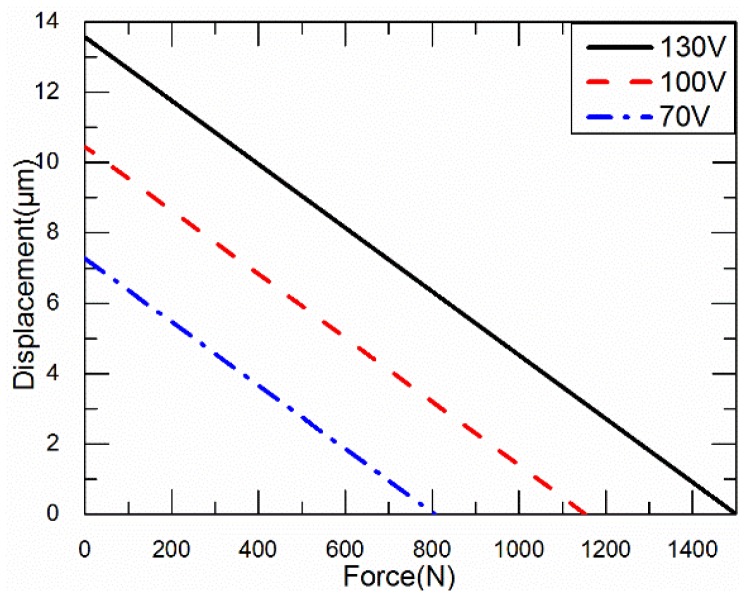
Relationship between the force and displacement under different working voltages.

The relationship between the force and displacement under different working voltages is shown in Figure 4.

When the load is constant, the larger the operating voltage, the larger the output displacement of the piezo stacks. When the displacement is constant, the output of the piezo stacks is positively correlated with the operating voltage. When the operating voltage of the piezo stacks is constant, the output displacement of the piezo stacks is negatively correlated with the load. As the load increases, the output displacement decreases. The piezo stacks have the same stiffness under different operating voltages.

When the working voltage of the piezo stacks is 130 V, the relationship between the displacement *S* (μm) and the force *F* (N) is shown in the Equation (1),

(1) S=−0.0088F+13.57,

Since the motion of piezo stacks is equivalent to the forced vibration of a damped single degree of freedom system, its dynamic analysis can be carried out according to that of the forced vibration of a damped single degree of freedom system.

In this paper, three piezo stacks are connected in series, whose total length is 54 mm. Since it is symmetrical, only half of the structure is needed to establish the static models. Then, the following Equation (2) can be obtained for piezo stacks,

(2) $$m_{e1}\ddot{x}+c_{1}\dot{x}+K_{V}x=K_{V}x_{0}U-F_{0}-F_{1},$$

where *m_e_*_1_ *= m*_1_/2 is the dynamic equivalent mass of the piezo stack. *m*_1_ is the mass of the piezo stack. *c*_1_, *K_V_*, and *x* are the damping coefficient, the stiffness and the displacement of the piezo stack respectively. *F*_1_ and *F*_0_ are the blocked force and preload on the piezo stack *U* is the voltage applied and *x*_0_ is the free displacement of the piezo stacks under a voltage unit.

### 3.2. Mathematical Model of the Displacement Output System

The diamond amplifier has two degrees of freedom in horizontal and vertical direction. First, the dynamic analysis of the jet dispenser is carried out only from the force analysis. It is simplified according to the analysis of double degree of freedom. Second, considering that there is a certain multiplier relationship between the horizontal and vertical displacement of the diamond amplifier, the force in the horizontal direction is equivalent to the force in the vertical direction; then, only the vertical motion needs to be analyzed. The analysis is simplified into a single degree of freedom system dynamics analysis.

#### 3.2.1. Double Degree of Freedom Output System

The displacement of the amplifier consists of two directions: horizontal and vertical. Through the amplifier, the input displacement in the horizontal direction is amplified and output in the vertical direction.

Because the diamond amplifier is elastomer, it can be analyzed according to the spring-mass-damper system, then its dynamic equation can be obtained.

The diamond amplifier is affected by *F_H_* horizontally and *F_V_* vertically. The output displacement is linear superposition of the displacement caused by *F_H_* and *F_V_*. The relationships between *x*, *y* and *F_H_*, *F_V_* are shown in Equations (3) and (4).

(3) $$x=\frac{F_{H}}{K_{1}}-\frac{F_{V}}{K_{2}^{\prime }},$$

(4) $$y=\frac{F_{H}}{K_{1}^{\prime }}-\frac{F_{V}}{K_{2}},$$

where *K*_1_ and *K*_2_ are the stiffness of the amplifier in the horizontal direction and vertical direction, respectively. *K′*_1_ is the displacement coefficient affected by *F_H_* in vertical direction. *K′*_2_ is the displacement coefficient affected by *F_V_* in horizontal direction.

Then, Equations (5) and (6) can be obtained:

(5) $$F_{H}=a_{1}x-b_{1}y,$$

(6) $$F_{V}=a_{2}x-b_{2}y,$$

When the mass of the amplifier cannot be neglected, the force analysis of the amplifier and the equivalent mass of the ejector elastic system can be used to obtain the dynamic Equation (7).

(7) $$m_{e2}\ddot{y}+c_{2}\dot{y}+K_{2}y=F_{V},$$

**Figure 5:**
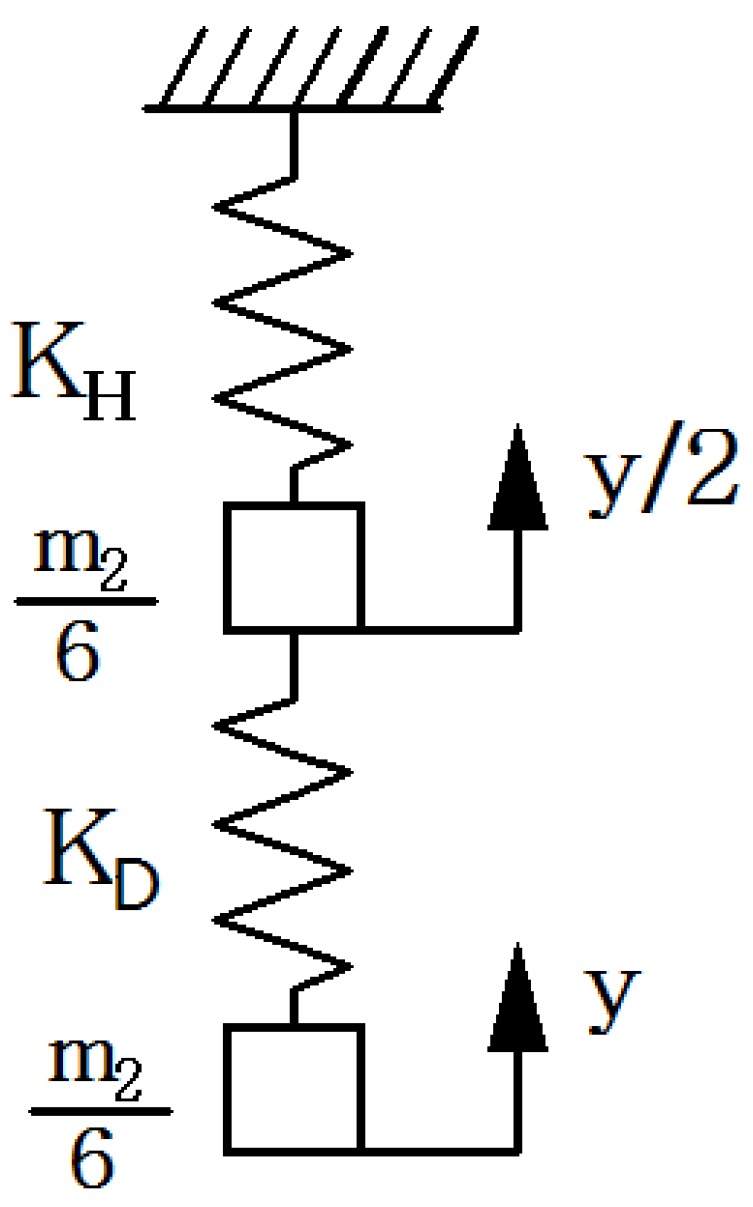
Spring-mass diagram of the amplifier.

Because of the symmetry of the diamond amplifier, its vertical direction can be equivalent to a spring-mass system, as shown in Figure 5.

From Figure 5 we can see that *K_H_* = *K_D_* = 2 × *K*_2_.

**Figure 6:**
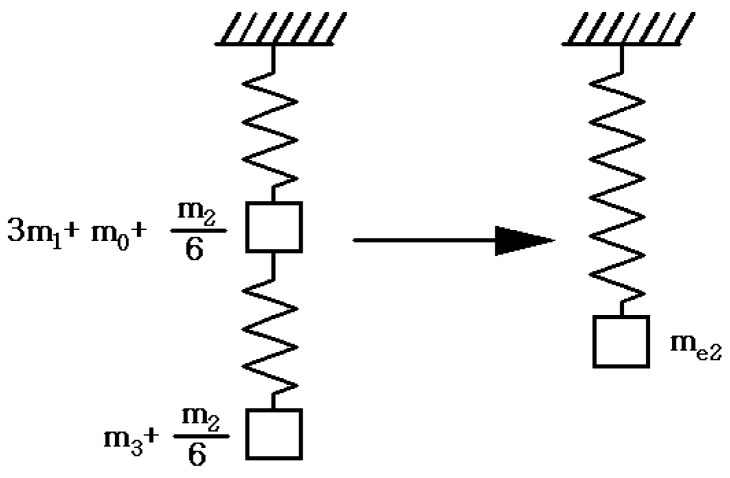
Spring-mass-damper diagram.

From Equations (2) and (5)–(8) can be obtained,

(8) $$\left\{ \begin{array}{c} m_{e1}\ddot{x}+c_{1}\dot{x}+K_{V}x=K_{V}x_{0}U_{0}-F_{0}-F_{1}\\ m_{e2}\ddot{y}+c_{2}\dot{y}+K_{2}y=F_{V}\\ F_{V}=a_{2}x-b_{2}y\\ F_{1}=K_{1}x \end{array}\right.,$$

where *c*_2_ is damping coefficient of the displacement output system. The equivalent mass of the vertical spring-mass-damper system includes the equivalent mass of the diamond amplifier in the vertical direction, the mass of the piezo stacks, the mass of the wedge and the mass of the needle. The simplified spring-mass-damper diagram is shown in Figure 6.

The equivalent mass of the vertical spring-mass-damper system is shown in the Equation (9).

(9) $$m_{e2}=\frac{3m_{1}+m_{0}+m_{2}/6}{2}+m_{3}+\frac{m_{2}}{6},$$

The equivalent mass of half of the piezo stacks is shown in the Equation (10).

(10) $$m_{e1}=\frac{m_{1}}{2},$$

where *m*_2_, *m*_3_, *m*_0_ are the mass of the diamond amplifier, the mass of the needle and the mass of the wedge respectively.

#### 3.2.2. Single Degree of Freedom Output System

The material of diamond amplifier used in this experiment is 65 Mn. In this study, the key geometric and material parameters of the diamond amplifier are listed in Table 2.

The diamond amplifier can be considered as a single degree of freedom elastic system.

**Figure 7:**
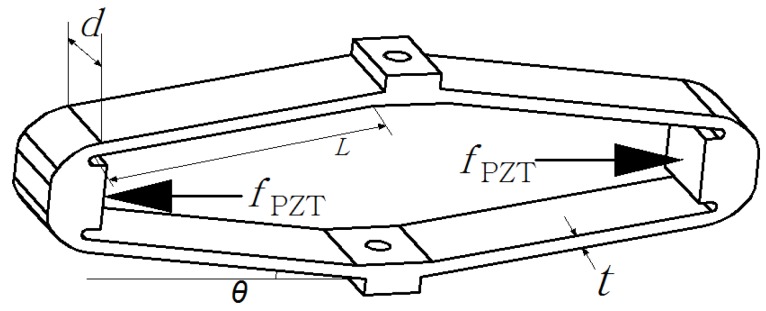
Diamond amplifier.

The diamond amplifier is depicted in Figure 7.

**Figure 8:**
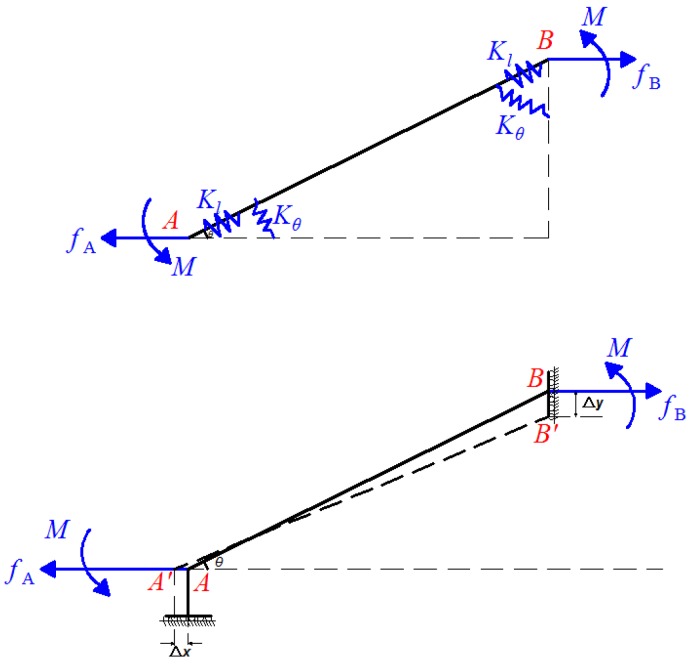
Force diagram of one arm of the diamond amplifier.

Since the symmetrical of the diamond amplifier, only a quarter of the structure is needed to establish the static model, as shown in Figure 8.

Considering the force equilibrium along the x-axis and the moment equilibrium, the following relations can be obtained:

(11) $$f_{A}=f_{B}=f=f_{PZT}/4,$$

(12) 2M=fLsinθ,

where *M* is a supplemented moment to ensure that the deflection angles at the both ends of link AB remain zero. *L* and *θ* are structural parameters of the amplifier as shown in Figure 7.

According to the principle of conservation of energy, work done by *f_PZT_* is transformed into the bending potential energy and the tensile deformation energy. Then, the following Equation (13) can be obtained for the amplifier.

(13) $$\frac{1}{2}f\Delta x=\int_{0}^{L}\frac{f^{2}(x)}{2EA(x)}dx+\int_{0}^{L}\frac{M^{2}(x)}{2EI(x)}dx,$$

where Δ*x*, *M*(*x*) and *f*(*x*) denote the input displacements, the moments in the elastic beam and the axial tension respectively. *A*(*x*) and *I*(*x*) are the area and moment of inertia of the corresponding cross-section about the neutral axis. *E* is Young’s modulus.

Based on Hooke’s law, the following relation is obtained:

(14) $$f(x)=f\cos\theta =K_{l}\Delta l,$$

where *K_l_* and Δ*l* are, respectively, the translational stiffness and the axial tensile displacement of the amplifier.

The moment in the elastic beam at the end point *x* changes along the neutral axis, and it can be obtained as:

(15) M(x)=M−fxsinθ=fsinθ(L/2−x),

In view of Equations (13)–(15), the strain energy produced by bending deformation in the amplifier can be obtained as:

(16) $$\Delta x=(\frac{cos^{2}\theta }{K_{l}}+\frac{L^{2}\sin^{2}\theta }{12K_{\theta }})f,$$

where *K_θ_* is the rotational stiffness of the diamond amplifier.

From the Euler–Bernoulli beam theory, the bending equation of a flexure element is:

(17) $$\frac{d^{2}\eta }{dx^{2}}=\frac{M(x)}{EI(x)},$$

Then, the output displacement in the amplifier can be deduced as:

(18) $$\Delta y=\eta \cos\theta =\cos\theta \times \iint \frac{M(x)}{EI(x)}dx=\frac{L^{2}f\sin\theta \cos\theta }{12K_{\theta }},$$

In view of Equations (16) and (18), the displacement amplification ratio of the diamond amplifier can be obtained as:

(19) $$R=\frac{2\Delta y}{2\Delta x}=\frac{K_{L}\times L^{2}\times \sin\theta \times \cos\theta }{12K_{\theta }\cos^{2}\theta +K_{L}\times L^{2}\times \sin^{2}\theta },$$

According to the displacement amplification ratio of the amplifier, the parameters *x* and *y* can be connected by the Equation (20).

(20) y=2H=x×2R,

The motion of the vertical direction corresponds to the force in horizontal direction. In the vertical direction, the amplifier and the needle form a spring-mass-damper system which is subjected to an external force of *F = f_PZT_/R*.

The single degree of freedom displacement output system mathematical model is shown in Equations (21) and (22).

(20) $$m_{e}\ddot{y}+c_{2}\dot{y}+Ky=F,$$

(21) $$m_{e}\ddot{y}+c_{2}\dot{y}+K_{2}y=(K_{v}x_{0}U_{0}-F_{0}-K_{1}y/2R)/R,$$

### 3.3. Numerical Analysis Model

A pulse voltage is applied to the piezo stacks. The amplitude of the pulse voltage is 130 V, the high-level time is 6 ms and the frequency is 100 Hz. The dynamic characteristics simulation model of the piezo stack is established with Simulink model. The parameters of the simulation model are listed in Table 3.

## 4. Results and Discussion

### 4.1. The Results of the Jet Dispenser for the Uper Analysis

**Figure 9:**
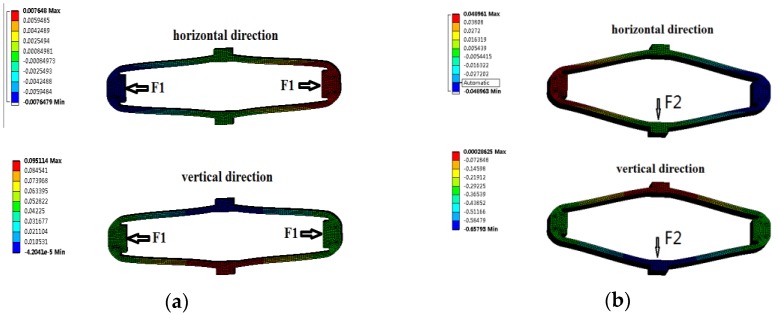
Diamond amplifier deformation: (**a**) Deformation under horizontal force; (**b**) Deformation under vertical force.

Static analysis of the amplifier was carried out by ANSYS. The magnifying mechanism is fixed at the top surface and its lower end is free in the vertical direction. *F_H_* and *F_V_* are applied in the horizontal and vertical directions respectively. The deformations of the amplifier are shown in Figure 9a,b.

**Figure 10:**
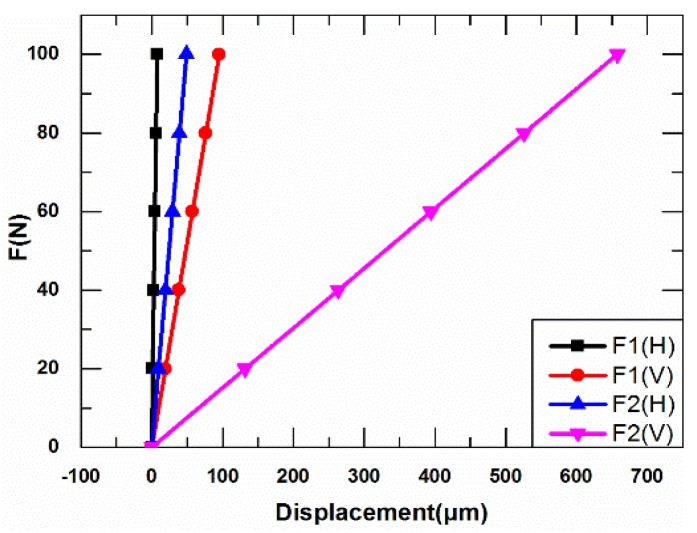
Relationship between the force and displacement.

Relationship between the force and displacement of the diamond amplifier is shown in Figure 10. The *K*_1_, *K′*_1_ and *K*_2_, *K′*_2_ can be obtained. The results are listed in Table 4.

The values of *K*_1_, *K′*_1_ and *K*_2_, *K′*_2_ in the Equations (5) and (6) can be obtained.

**Figure 11:**
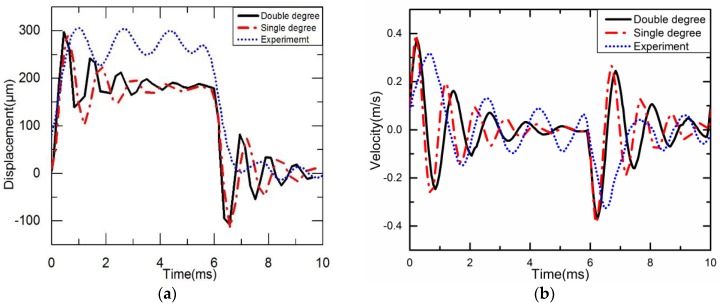
Dynamic characteristics of the needle: (**a**) The vertical displacement graph; (**b**) The vertical velocity graph.

According to Equations (8) and (22), Simulink is used to obtain the vertical displacement curves of the needle. The experimental method is used to get the displacement of the needle. A square wave voltage is applied. The voltage amplitude is 130 V, the time is 6 ms, and the frequency is 100 Hz. The displacement curves of the needle under double degree and single degree dynamic models and the experimental system in one cycle are shown in Figure 11a, and the velocity curves of the needle are shown in Figure 11b.

### 4.2. Another Piezo Jet Dispenser with New Diamond Amplifier Parameters

#### 4.2.1. Numerical Analysis Model

In order to make the results more reliable, a set of theoretical calculations and finite element simulations comparation under the changes of the amplifier’s parameters have been given as follows.

New geometric and material parameters of the diamond amplifier are listed in Table 5.

**Figure 12:**
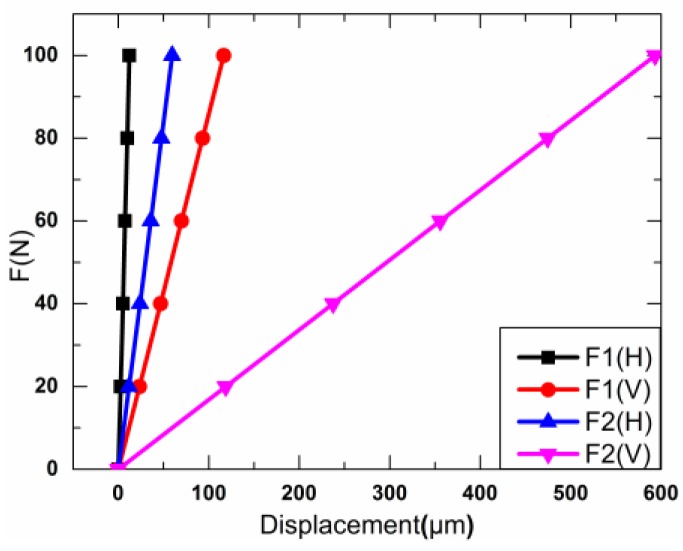
Relationship between the force and displacement.

Through the results and discussion in Section 3, the displacements of the new diamond amplifier under *F_H_* and *F_V_* are got and shown in Figure 12. The new *K*_1_, *K′*_1_ and *K*_2_, *K′*_2_ can be obtained. The results are listed in Table 6.

As a result, the values of *K*_1_, *K′*_1_ and *K*_2_, *K′*_2_ can be obtained in Equations (5) and (6), respectively.

#### 4.2.2. Dynamic Simulation Analysis of Piezo Jet Dispenser with a Diamond Amplifier

In the same experimental conditions as the previous example, the parameters of the new amplifier and the values of the obtained variables are substituted into the Equations (8) and (22). Simulink is used to obtain the vertical displacement curves of the needle under double degree and single degree dynamic models. Then, the vertical displacement curve of the piezoelectric driving dispenser is obtained through finite element analysis with Comsol.

**Figure 13:**
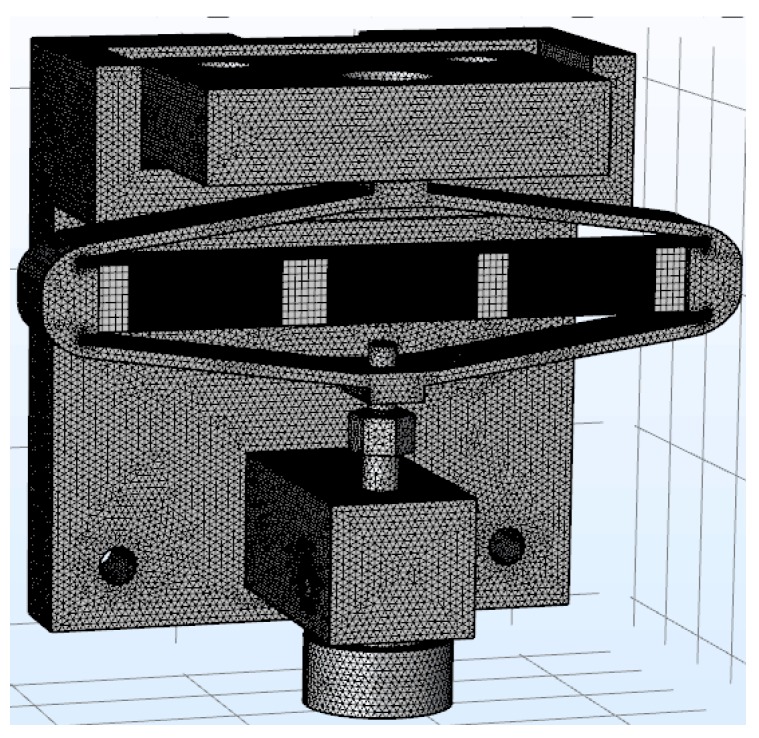
Mesh of the piezo jet dispenser with a diamond amplifier.

The mesh of the piezo jet dispenser with a diamond amplifier is shown in the Figure 13.

The multi physics field coupling simulation of the piezo jet dispenser with a diamond Amplifier is carried out by using the piezoelectric module of Comsol. With Comsol, the voltage function with time applied to piezoelectric ceramics in one cycle is approximate as the Equation (23).

(22) $$V(t)=\left\{ \begin{array}{c} 0\;(0\leq t<0.001)\\ 130000\times t-130\;(0.001\leq t<0.002)\\ 130\;(0.002\leq t<0.008)\\ -13000\times (t-0.008)+130\;(0.008\leq t<0.009)\\ 0\;(0.009\leq t<0.010) \end{array}\right.,$$

This is a pulse wave with a magnitude of 130, a high-level time of 6 ms, and a rise edge and fall edge time of 1 ms. By monitoring a point on the lower end of the amplifier within the waveform, the relationship between the displacement and speed of the lower end face with the time can be obtained.

**Figure 14:**
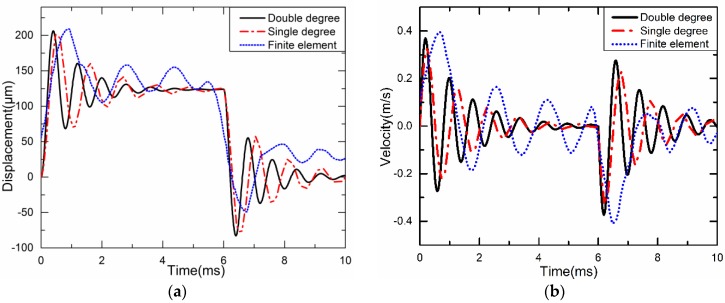
Dynamic characteristics of the needle: (**a**) The vertical displacement graph; (**b**) The vertical velocity graph.

The displacement curves of the needle under double degree and single degree dynamic models and the finite element analysis in one cycle are shown in Figure 14a. The velocity curves of the needle under double degree and single degree dynamic models and the finite element analysis in one cycle are shown in Figure 14b.

### 4.3. The Discussion

From Figure 11a, it can be seen that the dynamic analysis results of the double degree spring-mass-damper system are basically the same as those of the equivalent single degree spring-mass-damper system, and the maximum displacement can reach 300 μm. According to the experimental experience in the past, to obtain better jet performance, the needle needs to obtain a displacement of 200–300 μm [15]. Obviously, the diamond amplifier can meet the requirement because the maximum displacement under the experimental system is about 310 μm. The needle spends about 1.1 ms on single stroke, but the oscillation frequency of a curve obtained from experiments is not the same as the other two curves obtained from modeling. This is mainly due to the difference between the parameters of the piezo material, the thickness of the PZT, and the damping of the injection needle with the actual situation. However, the curves obtained from dynamic analysis are basically the same as those measured experimentally; this is reasonable and acceptable because the error between dynamic analysis and actual measurement is 5%. The experimental results agree with that from the simplified model, which proves that the simplified model is reliable. From the Figure 14a, it can be found that the dynamic analysis of the double degree spring-mass-damper system and the equivalent single degree spring-mass-damper system results are basically the same as those of the finite element analysis in one cycle, and the maximum displacement can reach 210 μm.

From Figure 11b, it can be seen that the maximum velocity reaches 0.39 m/s, 0.40 m/s and 0.31 m/s under double degree and single degree dynamic models and the experiment system, respectively. When the needle moves downward, its velocity of colliding with the nozzle slightly decreases to 0.41 m/s, 0.4 m/s and 0.38 m/s respectively. From Figure 14b, it can be seen that the maximum velocity reaches 0.37 m/s, 0.38 m/s and 0.40 m/s under double degree and single degree dynamic models and the experiment system respectively. When the needle moves upward, its velocity of colliding with the nozzle slightly decreases to 0.40 m/s, 0.39 m/s and 0.42 m/s respectively.

## 5. Conclusions

In summary, the mathematical models and simulation models have been established. The diamond amplifier is simplified as a single or double degree spring system while studying the dynamic characteristics. The tested results of the dynamic characteristics prove that the simplified method is reliable.

The most important and difficult problem in designing concerns the amplifier. The study provides a simplified method to design or assess diamond amplifier-based system. Through a little calculation, the feasibility of the diamond amplifier design can be verified. The method is also available for other similar piezo dispenser applications.

## Figures and Tables

**Table 1:** Piezo stacks technical parameters.

Size A × B × L (mm × mm × mm)	Maximum Displacement (μm/v)	Stiffness (N/μm)
7 × 8 × 18	0.1044	114

**Table 2:** Key geometric and material parameters of the diamond amplifier.

Parameters	Values
Flexure angle *θ* (°)	8
Flexure thickness *t* (mm)	1.4
Flexure breadth *d* (mm)	10
Flexure length *L* (mm)	30
*E* (Gpa)	211
Density *ρ* (kg/m^3^)	7820
Poisson’s ratio *b*	0.288

**Table 3:** Parameters of the dynamic model.

Parameters	Values
*m*_0_ (g)	8.6
*m*_1_ (g)	7.56
*m*_2_ (g)	25
*m*_3_ (g)	5.17
*C*_1_	120
*C*_2_	40.96
*F*_0_ (N)	140
*K_V_* (N/μm)	114

**Table 4:** Stiffness in the horizontal and vertical directions.

Direction	*K_H_*/N/μm	*K_V_*/N/μm
*F* *_H_*	13.04	1.05
*F* *_V_*	2.04	0.15

**Table 5:** Key geometric and material parameters of the diamond amplifier.

Parameters	Values
Flexure angle *θ* (°)	11
Flexure thickness *t* (mm)	1.4
Flexure breadth *d* (mm)	10
Flexure length *L* (mm)	30
*E* (Gpa)	211
Density *ρ* (kg/m^3^)	7820
Poisson’s ratio *b*	0.288

**Table 6:** Stiffness in the horizontal and vertical directions.

Direction	*K_H_*/N/μm	*K_V_*/N/μm
*F_H_*	8.18	0.86
*F_V_*	1.68	0.17
